# Endotoxin Disrupts Circadian Rhythms in Macrophages via Reactive Oxygen Species

**DOI:** 10.1371/journal.pone.0155075

**Published:** 2016-05-11

**Authors:** Yusi Wang, Paramita Pati, Yiming Xu, Feng Chen, David W. Stepp, Yuqing Huo, R. Daniel Rudic, David J. R. Fulton

**Affiliations:** 1 Vascular Biology Center, Medical College of Georgia at Augusta University, Augusta, Georgia, United States of America; 2 Department of Pharmacology, Medical College of Georgia at Augusta University, Augusta, Georgia, United States of America; 3 Department of Forensic Medicine, Nanjing Medical University, Nanjing, Jiangsu, China; McGill University, CANADA

## Abstract

The circadian clock is a transcriptional network that functions to regulate the expression of genes important in the anticipation of changes in cellular and organ function. Recent studies have revealed that the recognition of pathogens and subsequent initiation of inflammatory responses are strongly regulated by a macrophage-intrinsic circadian clock. We hypothesized that the circadian pattern of gene expression might be influenced by inflammatory stimuli and that loss of circadian function in immune cells can promote pro-inflammatory behavior. To investigate circadian rhythms in inflammatory cells, peritoneal macrophages were isolated from *mPer2*^luciferase^ transgenic mice and circadian oscillations were studied in response to stimuli. Using Cosinor analysis, we found that LPS significantly altered the circadian period in peritoneal macrophages from *mPer2*^luciferase^ mice while qPCR data suggested that the pattern of expression of the core circadian gene (*Bmal1*) was disrupted. Inhibition of TLR4 offered protection from the LPS-induced impairment in rhythm, suggesting a role for toll-like receptor signaling. To explore the mechanisms involved, we inhibited LPS-stimulated NO and superoxide. Inhibition of NO synthesis with L-NAME had no effect on circadian rhythms. In contrast, inhibition of superoxide with Tempol or PEG-SOD ameliorated the LPS-induced changes in circadian periodicity. In gain of function experiments, we found that overexpression of NOX5, a source of ROS, could significantly disrupt circadian function in a circadian reporter cell line (U2OS) whereas iNOS overexpression, a source of NO, was ineffective. To assess whether alteration of circadian rhythms influences macrophage function, peritoneal macrophages were isolated from *Bmal1*-KO and *Per*-TKO mice. Compared to WT macrophages, macrophages from circadian knockout mice exhibited altered balance between NO and ROS release, increased uptake of oxLDL and increased adhesion and migration. These results suggest that pro-inflammatory stimuli can disrupt circadian rhythms in macrophages and that impaired circadian rhythms may contribute to cardiovascular diseases by altering macrophage behavior.

## Introduction

In mammals, the master or central circadian clock is located in the hypothalamic suprachiasmatic nucleus (SCN) [1] of the brain and is synchronized by the light cycle. Clocks outside the brain, peripheral clocks, also interact with the central clock, but are also regulated by other signals aside from the light cycle. A commonality among central and peripheral clocks is the molecular components of the core circadian clock. The circadian clock is comprised of interdependent transcriptional/translational feedback loops. In the core loop, the CLOCK/BMAL1 heterodimer binds to E-box-sequences in promoter regions to activate transcription of clock-regulated genes that include the periods (*Per*) and cryptochromes (*Cry*) genes. The increased expression of PER and CRY proteins provide negative feedback by inhibiting CLOCK/BMAL1 activity until their levels are reduced. In an auxiliary loop, CLOCK/BMAL1 also controls the transcription of ROR and REV-ERB nuclear receptors, which bind to RRE elements in the *Bmal1* promoter and regulate BMAL1 expression. The net result of these feedback loops is oscillating patterns of gene expression and rhythmic changes in cell and organ physiology [2]. An estimated 43% of protein coding genes and 1000 non-coding RNAs have been proposed to undergo circadian changes in expression [3].

The cardiovascular system is subject to circadian regulation. One of the best known examples is the daily oscillation of blood pressure which in humans, peaks during the day and dips at night[4, 5]. These rhythms are important to the health of the organism and have evolved to anticipate the timing of physiological demands. Disruption of circadian rhythms through changes in environmental cues leads to increased cardiovascular diseases in animals and correlates with increased risk of death in humans[6]. The occurrence of heart attacks and strokes exhibit a daily rhythm[7] and the importance of the relationship between cardiovascular disease and circadian rhythms can be directly demonstrated in mice with genetic disruption of the circadian clock which results in impaired endothelium-dependent relaxation [8], exaggerated vascular remodeling [9, 10], altered cardiac ischemia reperfusion [11] increased atherosclerosis [12, 13], and impaired blood pressure control [14]. Thus peripheral clocks located in organ components of the cardiovascular system, such as the heart [15, 16], kidneys, [17] and blood vessels [10] exert an important role to regulate local physiology.

Chronic inflammation is believed to underlie many cardiovascular diseases including atherosclerosis, systemic and pulmonary hypertension and diabetes-induced vascular dysfunction. What is less well known is that inflammatory diseases exhibit a strong diurnal variation. Many aspects of the immune system exhibit daily fluctuations including the levels of cytokines, immune cell number and function and the expression of adhesion molecules [18]. Rheumatoid arthritis is characterized by a 24h rhythm of circulating concentrations of IL-6 [19] and both adaptive and innate immune responses are governed by circadian timing [20, 21]. While central mechanisms have been proposed to regulate clock function and timing in peripheral tissues [22], peripheral signals are also important. Immune cells may respond to numerous environmental cues in order to anticipate changes in function [23]. Macrophages, a key component of immune responses, exhibit robust circadian oscillations in gene expression, including TNFα[24], a master regulator of inflammation, while molecular disruption of circadian rhythms abolishes the rhythmic release of cytokines in *Bmal1* or *Rev-Erbα* knockout mice [25].

LPS is the major component of the outer membrane of Gram-negative bacteria and is composed of glycosylated lipid macromolecules with molecular weights ranging from 10–20 kDa. LPS is a potent pro-inflammatory molecule that elicits most of its effects via the extracellular TLR4 receptor [26]. Bacterial infections and LPS have been shown to stimulate macrophage migration, apoptosis [27–29] and also to accelerate atherosclerosis [30, 31]. LPS is intimately connected with circadian timing and studies have shown that the degree of cytokine release from immune cells and severity of endotoxic shock depends on the time of day that LPS is administered [24, 32] and that LPS can suppress the expression of circadian clock genes [33, 34]. However, the mechanisms by which LPS regulates macrophage clock function and the impact of the circadian clock on macrophage cell function are incompletely understood and were the major goals of this study.

## Materials and Methods

### Animals

All experiments were conducted in accord with the National Institutes of Health (NIH) Guide for the Care and Use of Laboratory Animals and approved and monitored by the Augusta University Institutional Animal Care and Use Committee (Augusta, GA). Studies were performed on 4- to 6-month-old male littermate control (wild-type, WT), *Per* triple (*Per1*, *Per2* and *Per3* KO, *Per*-TKO) and *Bmal1* KO, and *mPer2*^luciferase^ knock in mice as indicated. *Bmal1* KO and *mPer2*^luciferase^ were obtained from Jackson laboratories. *Period* isoform triple knockout mice (*Per-*TKO) were provided to us by Dr. David Weaver) and raised to a colony in our laboratory. These mice were originally generated by gene targeting in 129sv embryonic stem cells followed by chimeric males bred to isogenic 129/sv females[35].

### Peritoneal Macrophage Isolation

Macrophages were isolated as previously described [36]. In brief, *mPer2*^luciferase^, *Bmal1*-KO, *Per*-TKO and WT mice were injected with 1ml of 3% Brewer thioglycollate medium into the peritoneal cavity. Peritoneal macrophages were harvested 4 days after injection. The concentration of cells in the harvest medium was adjusted to 2 × 10^6^ total peritoneal cells/ml and cells were seeded into 96-well plates. Cells were allowed to adhere to the tissue culture plates for 2hr at 37°C. Non adherent cells were removed by gently washing three times with warm PBS.

### Measurement of Superoxide (ROS)

Peritoneal macrophages were seeded into 96-well plates and treated with or without LPS. 24 h later, the media was changed from RPMI to phenol-free Dulbecco’s modified Eagle’s medium (Sigma) containing L-012 (400μM, Wako) and incubated for 30 min prior to the addition of agonists. Luminescence was quantified over time using a Lumistar Galaxy (BMG) luminometer. The specificity of L-012 for ROS was confirmed in by co-incubation with the superoxide scavenger, SOD (100U/ml) as previously described [37]. Superoxide was also measured in COS-7 cells transfected with an active (*Nox5*β) or inactive NADPH oxidase (*Nox*5, H268L) using L-012-mediated chemiluminescence. Background signals were subtracted from control (*LacZ*) transected cells [38].

### Measurement of Nitric Oxide

Nitric oxide (NO) production was measured over time from macrophages or transfected cells via the accumulation of nitrite in cell culture medium using NO-specific chemiluminescence (280i NO analyzer, Ionics). COS-7 cells were transfected with either a control gene (*LacZ*) or iNOS. Background levels of nitrite in COS-7 cells expressing *LacZ* were subtracted from all measurements. In macrophages, the contribution of nitric oxide synthases (iNOS) to overall nitrite production was determined using the selective nitric oxide synthase inhibitor, L-NAME (400μM).

### Measurement of Circadian Rhythms

Peritoneal macrophages from *mPer2*^luciferase^ transgenic mice (1×10^4^ per well) were seeded into 96-well plates (white) and maintained in culture for 24h. U2OS *Bmal1*^dLuc^ cells were provided as a gift from Dr. John Hogenesch. The culture conditions were described previously [39]. Briefly, U2OS cells (human osteosarcoma cell line) harboring the Bmal1^*dLuc*^ circadian reporter were grown in regular DMEM supplemented with 10% FBS and antibiotics. Cells expressing *mPer2*^luciferase^ and Bmal1^*luciferase*^ were synchronized using 50% horse serum shock for 2h. The media was then changed to a luminescence buffer (0.1 mM luciferin containing medium) with or without the indicated treatments and bioluminescence recorded every 2 hours in a Lumistar Galaxy (BMG) luminometer maintained at 37°C. Oscillation curves were analyzed using the Cosinor program.

### Circadian Reporter Assays

The mouse *Per*1 promoter luciferase [40] was co-transfected with myc-*Bmal1* and HA-C*lock* with or without *Nox*5, inactive *Nox*5 or *iNOS* with and without L-N-NAME. Relative luciferase activity was measured using a Lumistar Galaxy (BMG) luminometer. Cell viability was determined using the CellTiter-Glo Assay from Promega.

### Analysis of Gene Expression

Total RNA was isolated from macrophages using TRIZOL and direct-zol (Zymo). cDNA was synthesized using the iScript cDNA Synthesis Kit (Bio-Rad) and relative gene expression using real time RT-PCR (Bio-Rad iQ SYBR Green) using the following primers. The sequences of the following primers were verified in previous publications and *Gapdh* or *18S* were used as control genes for all experiments as indicated (see Table 1).

**Table 1:** Primers used for real time PCR analysis and respective gene targets.

Gene	Sense	Antisense	Accession#	Ref.
*Bmal1*	TTCTCCAGGAGGCAAGAAGA	TTGCTGCCTCATCGTTACTG	NM_007489	[41]
*Tnfα*	CGTCAGCCGATTTGCTATCT	CGGACTCCGCAAAGTCTAAG	M11731	[42]
*Il6*	ACAACCACGGCCTTCCCTACTT	CACGATTTCCCAGAGAACATGTG	NM_031168	[43]
*18S*	CTTAGAGGGACAAGTGGCG	ACGCTGAGCCAGTCAGTGTA	NR_003278	[44]
*Gapdh*	ACCCAGAAGACTGTGGATGG	CACATTGGGGGTAGGAACAC	M32599	[45]

### In Vitro Foam Cell Assays

Peritoneal macrophages were isolated from the indicated mouse models and exposed to oxLDL (50μg/ml) for 72h. Cells were fixed with 4% paraformaldehyde in PBS for 10 min and washed twice with PBS, followed by 60% 2-propanol for 2 min prior to staining with 0.2% oil red O (Sigma) in 60% 2-propanol for 10 min. Slides were then washed with 2-propanol and PBS. Cholesterol ester and free cholesterol content of macrophages were determined using the Cholesterol Assay Kit (Cayman Chemical Company).

### Macrophage Migration Assays

Peritoneal macrophage migration assays were described previously [46]. Cells were seeded in each well of Oris plate (Platypus Technologies, Madison, WI, USA). The inserted column were removed carefully after 24h until the seeded cells attached to the plate. Inserts were then carefully removed and cells gently washed with warm PBS. The cells were incubated with or without LPS (100 ng/ml) in RPMI medium for 24 h and then stained with 5 *μ*M of calcein AM for 30 min. BMG Galaxy fluorescent microplate reader was used to measure excitation/emission wavelengths (485/515 nm) of migrated cells into the previously restricted zone.

### Macrophage Adhesion Assays

Macrophage labeling and adhesion assays were performed as described previously [47]. Briefly, peritoneal macrophages were labeled with 5 *μ*mol/L of CFDA-SE in phosphate-buffered saline (PBS) at 37°C for 10 minutes and then washed three times with cell culture medium. 1×10^5^ peritoneal macrophages were then added to activated human aortic endothelial cells treated with 10 ng/mL TNFα overnight in 12-well plates. After incubation for 15 minutes at 37°C, cells were washed twice with PBS and the fluorescence signal was measured with a Lumistar Galaxy (BMG) plate reader.

### Statistical Analysis

Data are reported as mean ± SEM. Comparisons were made using a student’s t-test (pairwise) or ANOVA (one and two way) with a Bonferroni post-hoc test where appropriate (Prism). Oscillation curves were analyzed by cosinor as described previously and changes in amplitude and acrophase (timing of the peak response) were determined [35]. Differences were considered as significant at p < 0.05.

## Results

### LPS Alters Circadian Rhythms in Macrophages

**Fig 1:**
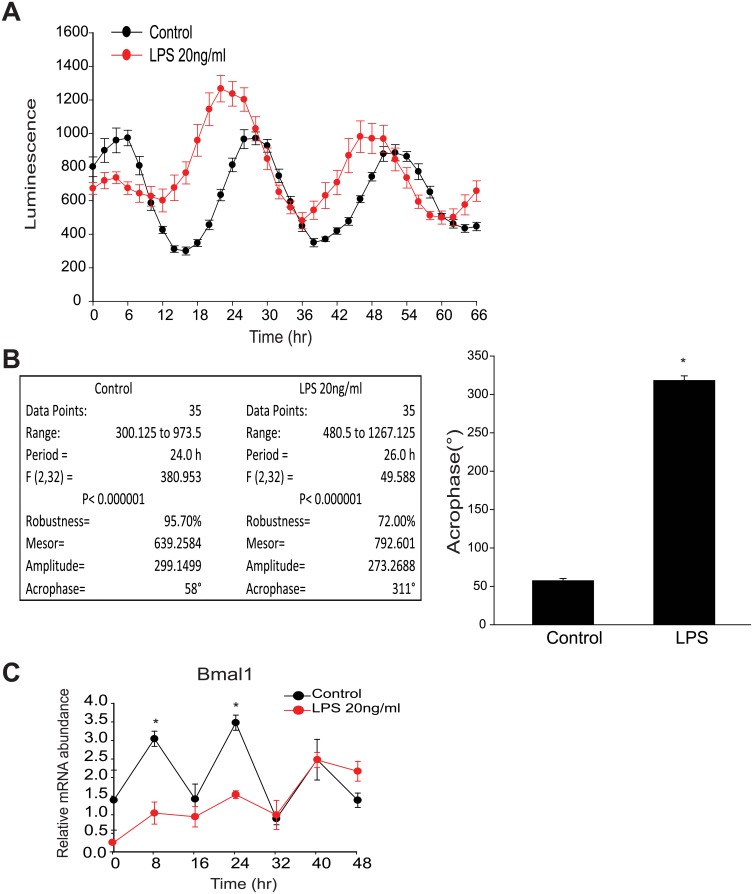
LPS induces a phase shift in synchronized peritoneal macrophages and impairs the expression of core circadian genes in peritoneal macrophages. **(A)** Peritoneal macrophages from *mPer2*^luciferase^ transgenic mice were seeded in 96-well plate (white) for 24 hours. After 2h serum shock, cells were kept in luminescence buffer in presence or absence of LPS (20ng/ml) and bioluminescence was recorded every 2 hours which starts from Time 0. **(B)** Oscillation curves were analyzed by cosinor (time 0 and 24h represented by 0° and 360°, respectfully) and acrophase determined (mean ± SEM, n = 8, t-test, * *p*<0.05, *versus* Control). **(C)** Peritoneal macrophages were synchronized and mRNA levels of *Bmal1* and 18S were assessed every 8h with qRT-PCR in presence or absence of LPS (20ng/ml). Transcript abundance (ΔΔCt) was reported relative to Time 0 in the control group (mean ± SEM, n = 5, one-way ANOVA with Bonferroni post hoc correction, *p<0.05, *versus* control).

**Fig 2:**
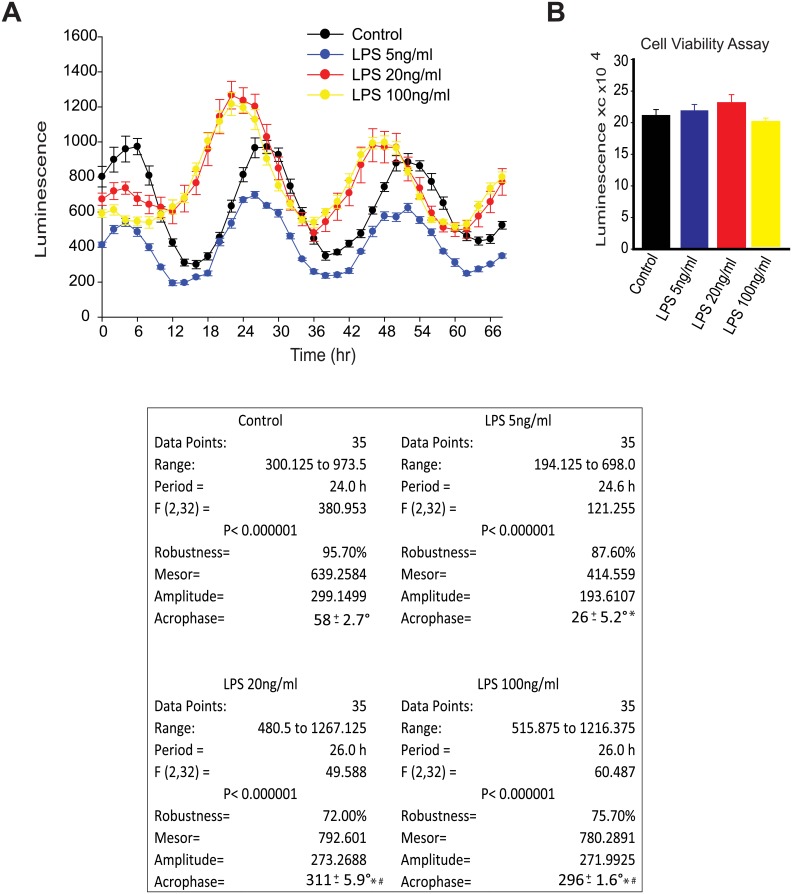
LPS promotes a circadian phase-shift at low concentrations. Varying doses of LPS (0, 5, 20 and 100ng/ml) were added into the luminescence buffer at Time 0 after serum shock. **(A)** *Left panel*: Bioluminescence were recorded every 2h continuing for 68 hours. *Right panel*: Relative cell numbers were measured via a cell viability assay at the end of the luminescence measurements (mean ± SEM, n = 4, one-way ANOVA, ns versus Control). **(B)** Oscillation curves were analyzed by cosinor and acrophase compared by one-way Anova with Bonferroni post hoc correction (mean ± SEM, n = 8, *p<0.05, *versus* control, # *p*<0.05, *versus* LPS 5ng/ml).

Previous studies have demonstrated the existence of a local circadian clock in peritoneal macrophages [24]. Thus, we undertook studies to investigate whether the circadian clock in macrophages can be regulated by pro-inflammatory stimuli by exposing peritoneal macrophages to LPS. After synchronizing macrophage clocks with serum shock, LPS was adminstered which promoted the disruption of circadian rhythms and dampened oscillation as determined by *mPer2*^luciferase^ activity (Fig 1A). More detailed analysis of circadian rhythm by cosinor revealed that the acrophase (a marker for phase shift obtained by measuring the extent and timing of changes within a cycle [48, 49]) was significantly altered by LPS in synchronized peritoneal macrophages, indicative of a phase shift (Fig 1B). Similar results were obtained using other stimuli i.e. dexamethasone to synchronize macrophage clocks. To obtain additional evidence of effects on clock function, we next assessed changes in the mRNA expression of *Bmal1* over time. qRT-PCR results demonstrated that LPS repressed mRNA expression of the core clock gene, *Bmal1* at multiple time points (Fig 1C). To explore the potency of the LPS-induced phase-shift, peritoneal macrophages were challenged with progressively lower doses of LPS. While LPS was able to induce a phase shift even at the lowest dose tested, this phase shift was potentiated at the higher doses tested (Fig 2A). The low dose of LPS slightly reduced circadian amplitude and acrophase versus higher doses of LPS which increase both more robustly. These effects were independent of an effect on cell viability, as assesment of relative ATP levels revealed no significant difference between control and LPS at the doses employed (Fig 2B).

### Antagonism of TLR4 reverses the LPS-induced phase-shift

**Fig 3:**
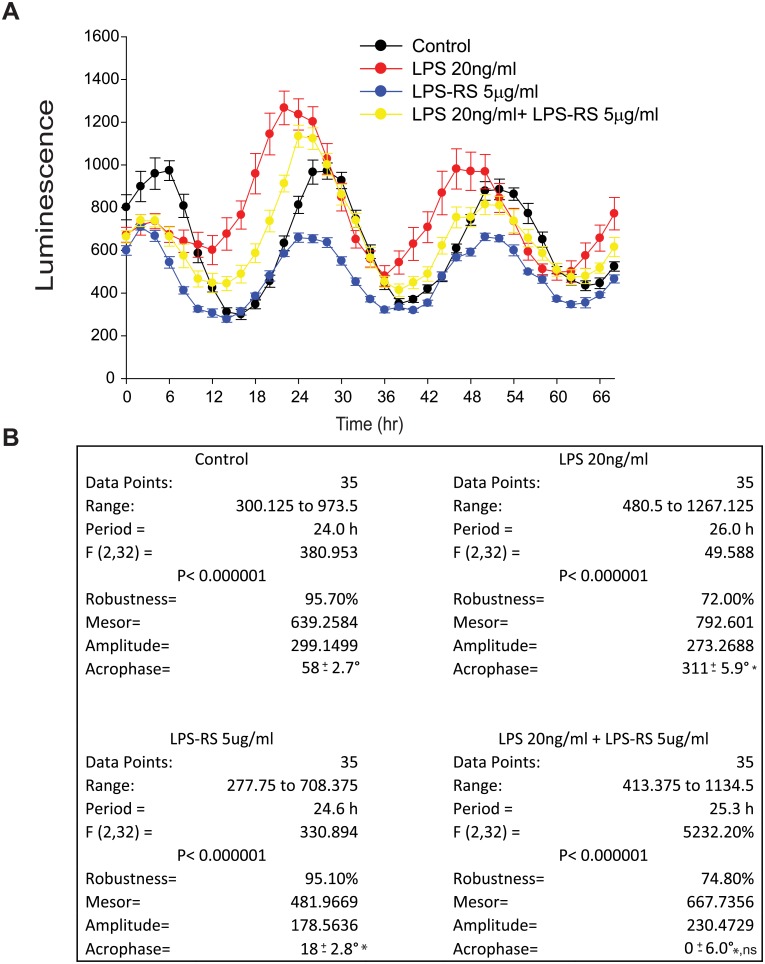
Inhibition of TLR4 reverses the LPS-induced phase-shift. Peritoneal macrophages were isolated from *mPer2*^luciferase^ transgenic mice and subjected to synchronization via dexamethasone shock. 20ng/ml LPS with or without LPS-RS (5μg/ml) was added to the luminescence buffer at Time 0. **(A)** Bioluminescence were recorded every 2h continuing for 68 hours. **(B)** Oscillation curves were analyzed by cosinor and acrophase compared by one-way Anova with Bonferroni post hoc correction (mean ± SEM, n = 8, *p<0.05, *versus* Control, ns *p*>0.05, *versus* LPS-RS).

Among the many TLR family members, TLR4 is regarded as the major cellular receptor for LPS [26]. To investigate the mechanism by which LPS causes circadian phase shift in macrophages, macrophages were treated with a TLR4 antagonist and then circadian rhythms assesses using *Per*2 promoter-dependent luminescence. Previous studies have shown that hypo-acylated LPS (LPS-RS) competitively antagonizes hexa-acylated LPS [50] by competing with the same binding site on MD-2 to represses TLR4 signaling [51]. In our studies, we found that LPS-RS prevented the phase-shift in response to LPS in peritoneal macrcophages (Fig 3A) as evidenced by a dramatic blunting of the shift in acrophase in response to LPS (Fig 3B). LPS-RS alone induced a slight shift in acrophase compared to control (Fig 3A and 3B).

### LPS stimulates NO and ROS release from peritoneal macrophages

**Fig 4:**
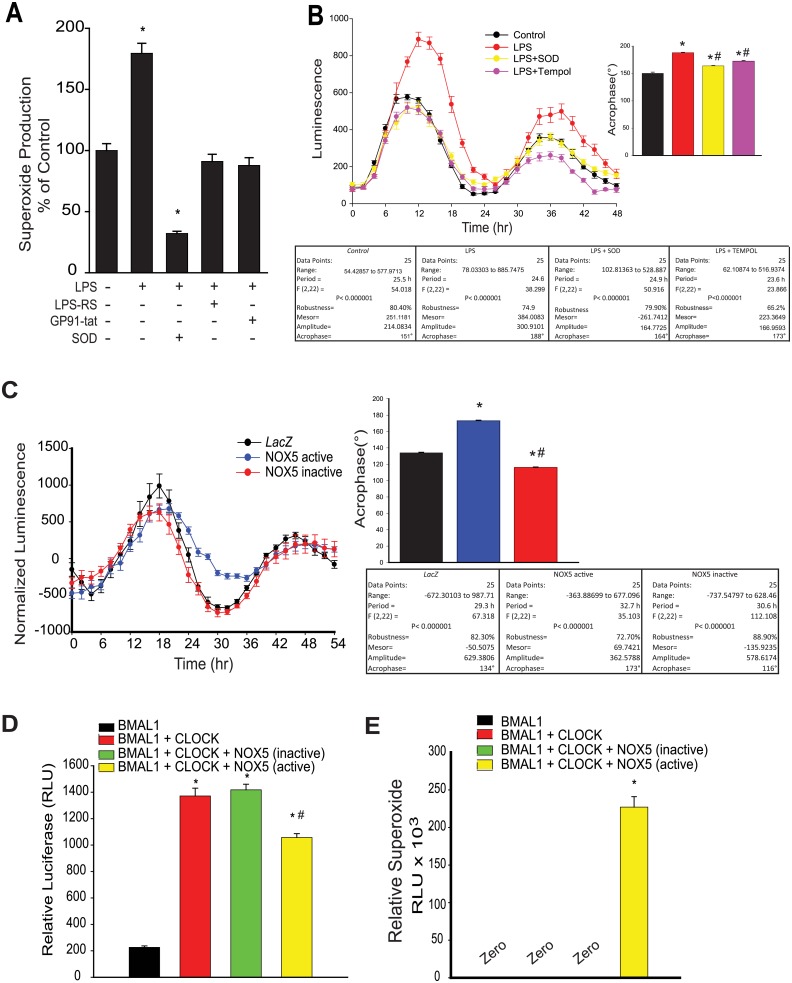
LPS stimulates ROS release from peritoneal macrophage and elevated ROS impairs the function of circadian transcription factors. **(A)** Peritoneal macrophages were isolated from *mPer2*^luciferase^ transgenic mice and subjected to different treatments over 24h (LPS 20ng/ml, LPS-RS 5μg/ml, gp91 ds-tat 1μM). Unstimulated or basal superoxide release was monitored using L-012 chemiluminescence (mean ± SEM, n = 5, one-way ANOVA with Bonferroni post hoc correction, *p<0.05, versus Control). **(B)** Peritoneal macrophages from *mPer2*^luciferase^ mice were subjected to different treatments (LPS 20ng/ml, PEG-SOD, 100U/ml, Tempol 0.4mM) and bioluminescence recorded every 2h for 68 hours (mean ± SEM, n = 5, acrophase were compared via one-way ANOVA with Bonferroni post hoc correction, *p<0.05, versus control. # p<0.05, versus LPS). **(C)** U2OS *Bma1*^luciferase^ cells were transduced with active or inactive *Nox5* adenovirus (15 MOI) and the oscillation of expressed luciferase activity recorded every 2h after serum shock. Oscillation curves were analyzed by cosinor and acrophase compared by one-way Anova with Bonferroni post hoc correction (mean ± SEM, n = 6, *p<0.05, versus Control, # p<0.05, versus Nox5 active). **(D)** *Per1* promoter transactivation was assessed by a dual luciferase assay in transfected COS cells expressing BMAL1, BMAL1+CLOCK in the presence or absence of the ROS generator NOX5 or an inactive NOX5 enzyme (H268Q), (mean ± SEM, n = 5, one-way ANOVA with Bonferroni post hoc correction, *p<0.05, versus *Bmal1* alone. # p<0.05, versus Nox5 active). **(E)** SOD-sensitive superoxide production was monitored by L-012 chemiluminescence. Results are presented as mean ± SEM, n = 6, one-way ANOVA with Bonferroni post hoc correction, *p<0.05, versus *Bmal1* alone.

Macrophages are a major source of ROS and increased production of ROS is a prominent outcome of activation of the LPS-TLR4 signaling pathway [52]. In peritoneal macrophages, LPS stimulated ROS production, and this was inhibited by the TLR4 antagonist LPS-RS as well as the selective NOX2 inhibitor (gp91 ds-tat) and superoxide dismutase (SOD) (Fig 4A). We next investigated whether this increased ROS mediates the ability of LPS to alter circadian rhythms. Scavengers of superoxide, superoxide dismutase (SOD) and Tempol reversed the LPS-induced circadian disruption (Fig 4B). To directly assess a relationship between ROS and altered circadian rhythms, we transduced a circadian reporter cell line (U2OS *Bmal1*^luciferase^) with NADPH oxidase 5 (*Nox*5) an enzyme that produces superoxide from a single gene product both at baseline and following calcium-dependent stimuli [37]. To distinguish between any possible effects of NOX5 per se versus the enzymatic product [53], U2OS cells were also transduced with adenoviruses encoding a mutant form of NOX5 (H268Q) that disrupts heme binding and the ability to produce superoxide. Circadian rhythms in U2OS cells expressing the active form of NOX5 were phase shifted compared to control, an effect that was absent in cells expressing the inactive NOX5 enzyme (Fig 4C). To determine how increased ROS might affect circadian oscillation, we next assessed the effect of ROS on circadian transcription factors in a promoter-dual luciferase reporter assay. In control transfected cells, co-transfection of *Bmal1* and *Clock* robustly transactivated the *mPer2*^luciferase^ reporter whereas in cells co-transfected with *Nox*5, *Bmal1* and *Clock*, promoter activation was reduced (Fig 4D). In contrast, the inactive NOX5 mutant was without effect on promoter activation. ROS production from control, active and inactive NOX5 constructs is shown in Fig 4E.

**Fig 5:**
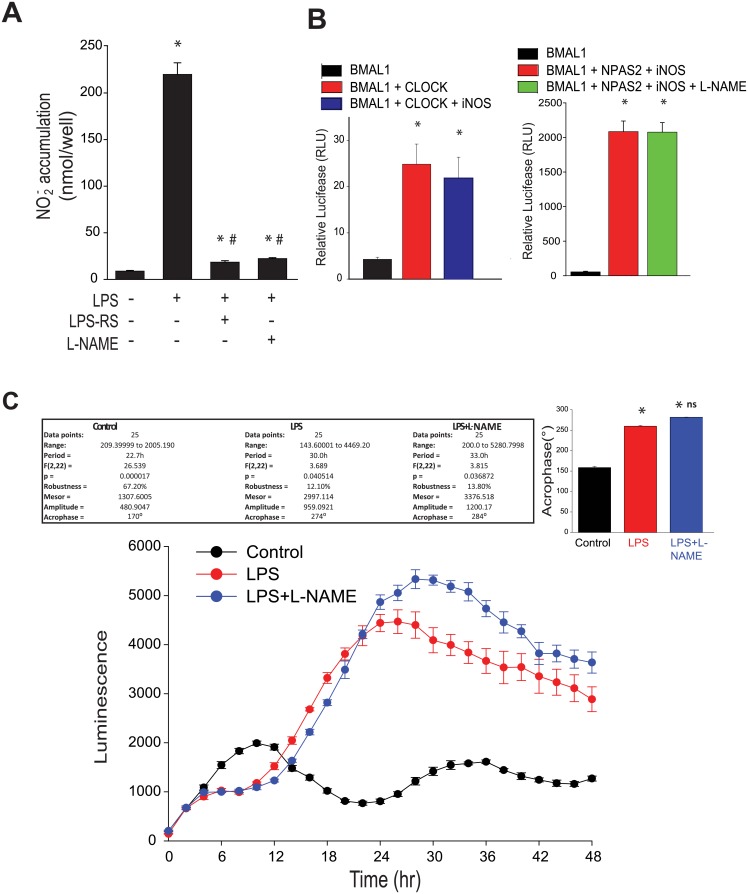
LPS increases NO release from peritoneal macrophage and iNOS-derived NO has no effect on circadian transcription factor activity. **(A)** NO release was measured by chemiluminescence detection of NO_2_^−^ (mean ± SEM, n = 5, one-way ANOVA with Bonferroni post hoc correction, *p<0.05, versus control. # p<0.05, versus LPS). **(B)** Dual luciferase assay in COS cells expressing the *Per*1 promoter luciferase and BMAL1 and CLOCK or BMAL1, BMAL1+NPAS2+iNOS in the presence or absence of L-NAME (2mM), mean ± SEM, n = 6, one-way ANOVA with Bonferroni post hoc correction, *p<0.05, versus Bmal1 alone). **(C)** Peritoneal macrophages were synchronized as described and exposed to LPS (20ng/ml) with or without L-NAME (2mM). Bioluminescence was recorded every 2h for 48 hours. Oscillation curves were analyzed by cosinor and acrophase compared by one-way Anova with Bonferroni post hoc correction (mean ± SEM, n = 5, *p<0.05, versus Control, ns versus LPS).

Another important mediator of inflammatory signaling in macrophages is nitric oxide (NO). The high output, inducible nitric oxide synthase (iNOS) was originally identified in macrophages as a major source of NO/nitrite. LPS is a potent inducer of iNOS expression and NO release in murine macrophages [54]. To investigate whether LPS and TLR4 inhibitors influence NO release in murine peritoneal macrophages, macrophages were treated with LPS and NO levels were measured by NO-specific chemiluminescence. Compared to control, LPS robustly induced NO production which was negated by the TLR4 antagonist, LPS-RS and the inhibitor of NOS, L-NAME (Fig 5A). To determine whether NO influences circadian transcription factor activity, we performed a dual promoter luciferase assay in COS-7 cells transfected with the *Per*1 promoter luciferase constructs and renilla luciferase, co-transfected with either *Bmal1*, *Bmal*1 and *Clock*, *Bmal*1 and *Clock* and iNOS (NO source) and *Bmal1* and *Npas2* and *iNOS* in the presence and absence of L-NAME. There was no significant effect of iNOS on BMAL1:CLOCK activity or iNOS-derived NO on BMAL1:NPAS2 activity (Fig 5B). Furthermore, in peritoneal macrophages isolated from *mPer2*^luciferase^ mice, the LPS-induced phase shift was not altered in the presence of L-NAME (Fig 5C).

### Peritoneal macrophages from circadian clock knockout mice exhibit altered NO and ROS release

**Fig 6:**
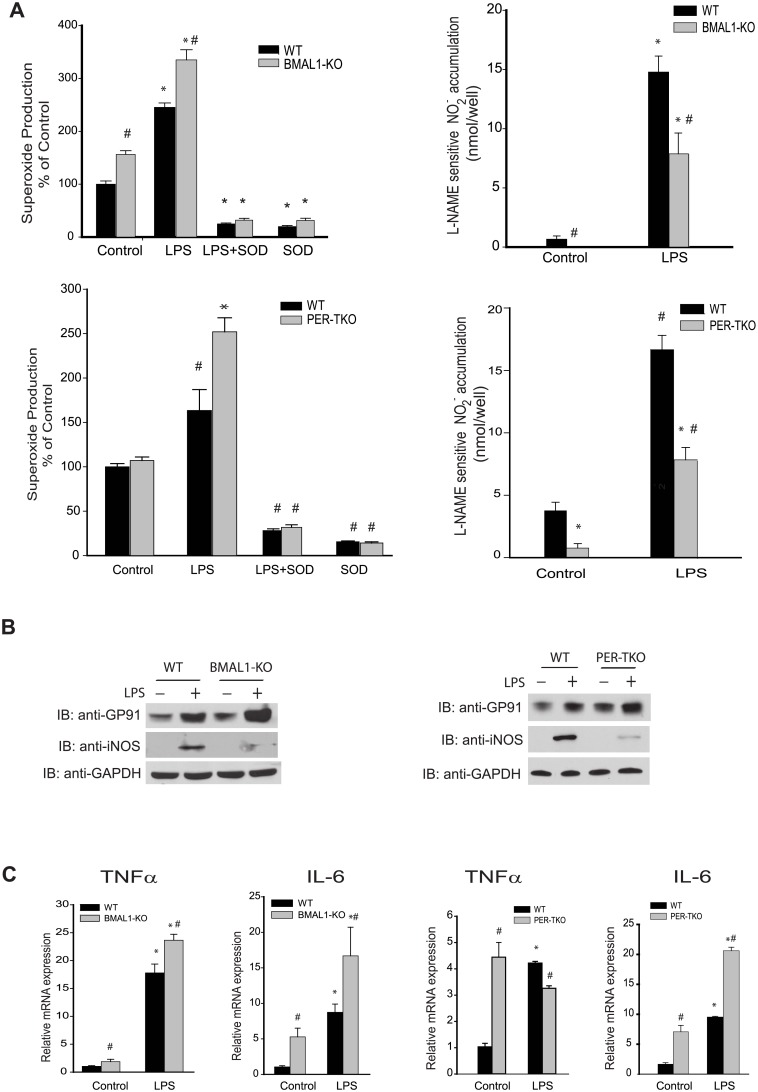
Circadian genes knockout alters the balance ROS and NO release from peritoneal macrophages. Peritoneal macrophages were isolated from WT mice and circadian gene knockout mice (*Bmal1*-KO mice and *Per*-TKO mice), and cells were subjected to different treatments over 24h. **(A)** Unstimulated or basal superoxide release was monitored using L-012. NO release was measured by NO-specific chemiluminescence of NO_2_^−^. The data was normalized by residual NO_2_^−^ detected in the presence of L-NAME (mean ± SEM, n = 6, two-way ANOVA with Bonferroni post hoc correction, *p<0.05, versus control. # p<0.05, versus WT). **(B)** Peritoneal macrophages were isolated from WT or circadian clock knockout mice (Bmal1 KO and Per-TKO), exposed to vehicle or LPS (20ng/ml, 24h) and lysed in Laemmli sample buffer. Cell lysates were subjected to SDS-PAGE and immunoblotted with antibodies to gp91phox (NOX2), iNOS and GAPDH. Results are representative of 3 experiments. **(C)** Peritoneal macrophages were isolated from WT or circadian clock knockout mice (*Bmal1* KO and *Per*-TKO), exposed to vehicle or LPS (20ng/ml, 24h) and lysed in TRIZOL for mRNA extraction. Relative mRNA expression levels of *Tnfα* and *Il-6* were measured by qRT-PCR (ΔΔCt) normalized to GAPDH (mean ± SEM, n = 6, two-way ANOVA with Bonferroni post hoc correction, *p<0.05 versus control, # p<0.05 versus WT).

To evaluate whether the loss of circadian rhythms influences macrophage behavior, we next measured NO and ROS release from macrophages isolated from WT and circadian gene knockout mice. Superoxide production from 2 different models of circadian clock disruption, *Bmal1*-KO and *Per*-TKO mice was consistently elevated following LPS challenge and higher in *Bmal1*-KO under basal conditions (Fig 6A, left panels). In contrast, NO release from circadian gene knockout mice was consistently decreased both at baseline and following LPS stimulated compared with WT mice (Fig 6A, right 2 panels). Analysis of protein expression by Western blot revealed increased gp91phox (NOX2) expression but decreased iNOS expression in circadian clock knockout mice (Fig 6B). To further investigate the pro-inflammatory consequences of circadian clock gene disruption, we extracted RNA from peritoneal macrophages and measured the relative expression of cytokines by real-time PCR. Both basal and LPS-stimulated *Tnfα* and *Il-6* mRNA expression were elevated in *Bmal*1-KO mice. While *Il*-6 was also elevated in *Per*-TKO mice under the same conditions, *Tnfα* mRNA levels were higher at baseline and unchanged following LPS in *Per*-TKO mice relative to WT mice (Fig 6C).

### Circadian clock disruption increases LDL uptake by macrophages

**Fig 7:**
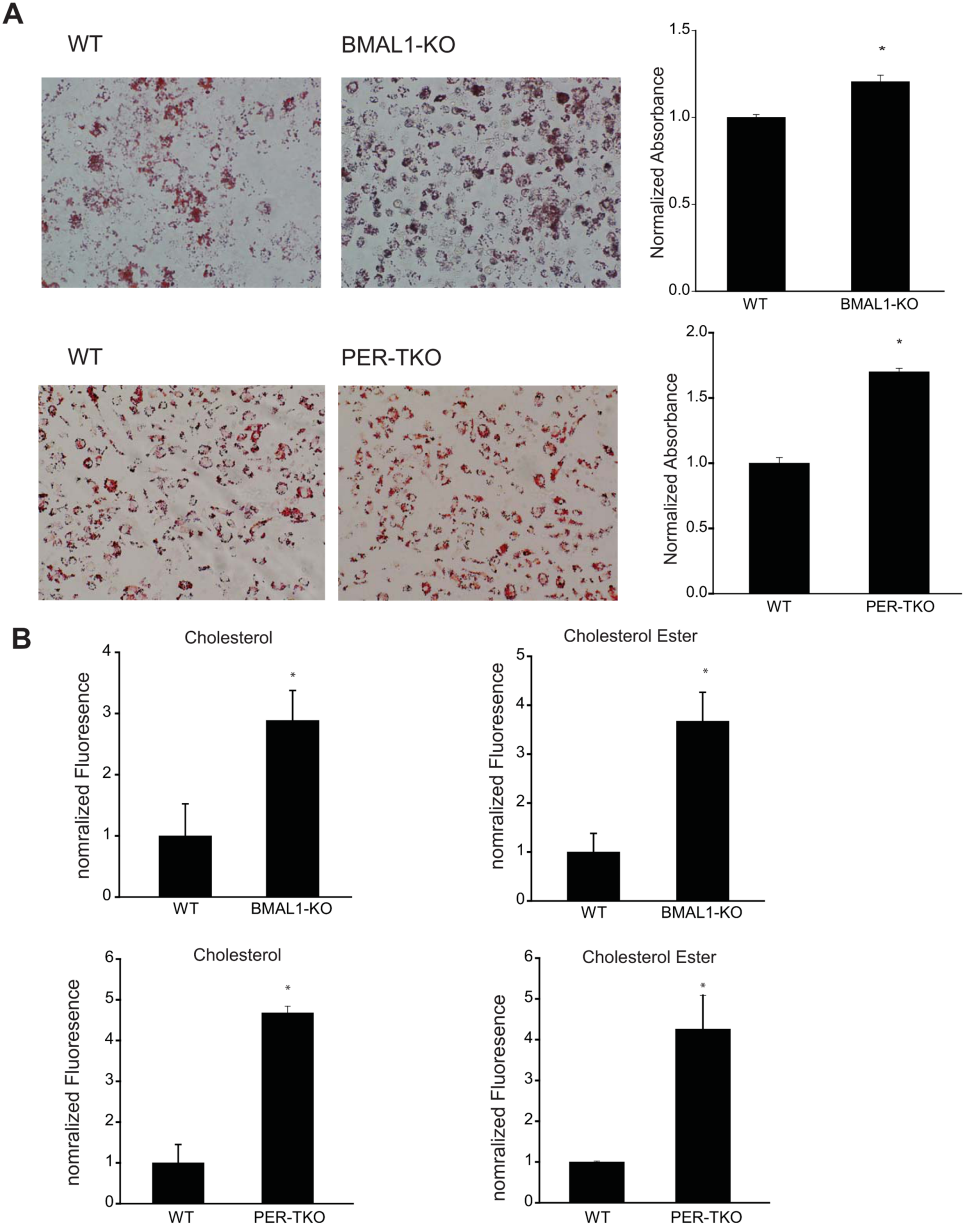
Circadian clock disruption increases LDL uptake by macrophages. Peritoneal macrophages were isolated from WT, *Bmal1* KO or *Per*-TKO mice and cells exposed to oxLDL (50μg/ml) for 72h. **(A)** Oil red O staining for lipid uptake in macrophages. The extent of staining was quantitated by measurement of fluorescent intensity. (mean ± SEM n = 4, t-test, *p<0.05 versus WT). **(B)** Measurement of cholesterol ester and free cholesterol content from macrophages. (mean ± SEM n = 4, t-test, *p<0.05 versus WT

**Fig 8:**
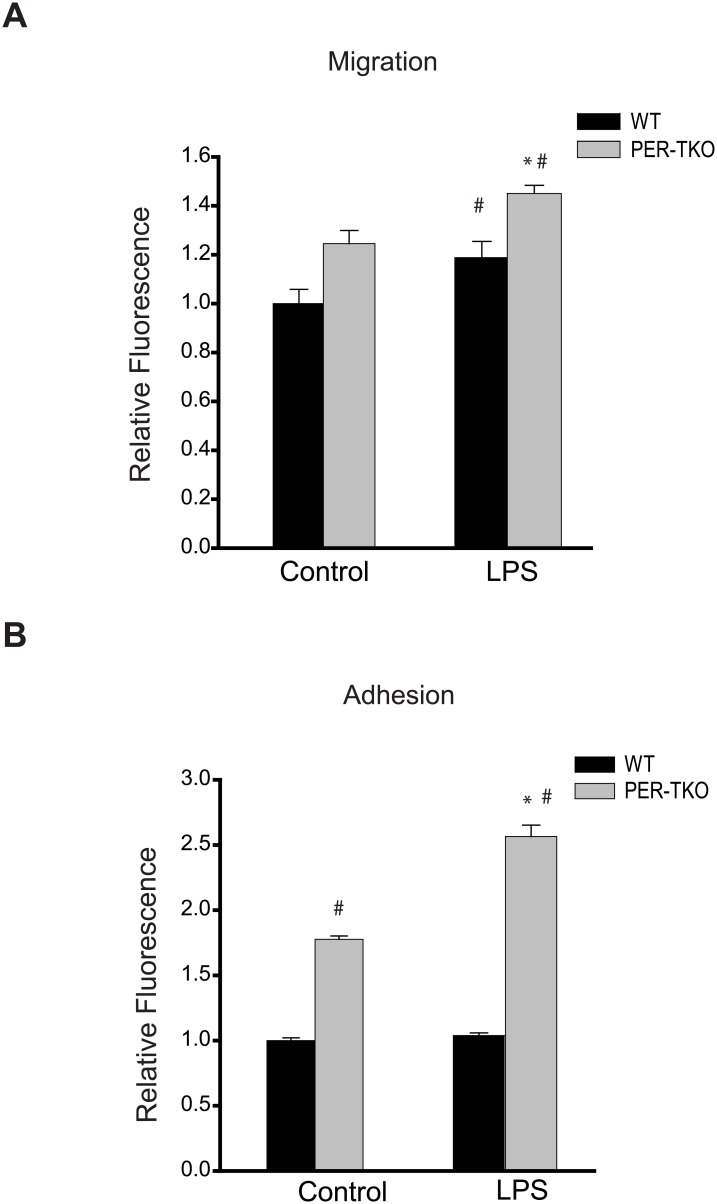
Circadian clock disruption alters the migration and adhesion of macrophages. **(A)** The effect of circadian disruption on macrophage migration was evaluated using the Oris Cell Migration Assay. Peritoneal macrophages were isolated from WT and *Per*-TKO mice and subjected to the indicated treatment (LPS 100ng/ml) for 24h, cells stained with calcein AM for 30 min and the fluorescence signal in the migration zone quantified. (mean ± SEM n = 4–5, two-way ANOVA with Bonferroni post hoc correction, *p<0.05 versus control, # p<0.05 versus WT). **(B)** Fluorescently labeled peritoneal macrophages were incubated with activated adherent human aortic endothelial cells for 15 minutes at 37°C and the degree of cell adhesion assay was quantified (mean ± SEM n = 6, two-way ANOVA with Bonferroni post hoc correction, *p<0.05 versus control, # p<0.05 versus WT).

Recent studies have reported that circadian clock-deficient mice have increased susceptibility to atherosclerosis [55]. Given the importance role of macrophages in the development of atherosclerosis, we next determined whether disruption of the circadian clock alters the ability of macrophages to take up lipids. Peritoneal macrophages from WT and circadian gene knockout mice were exposed to oxLDL for 72h and then assessed for lipid uptake via Oil Red O staining and measurement of cholesterol levels. As shown in Fig 7A, macrophages from both *Bmal1*-KO and *Per*-TKO have increased Oil Red O staining and increased levels of both cholesterol and cholesterol ester (Fig 7B). To explore whether circadian disruption alters other macrophage behaviors, we performed cell migration and adhesion assays separately. Fig 8A shows that cell migration was elevated in macrophages from *Per*-TKO mice at baseline and following LPS stimulation. We next examined whether circadian clock gene disruption could influence macrophage adhesion to endothelial cells. Both at baseline and following LPS stimulation, macrophages from *Per*-TKO mice exhibited about a 2 fold increase in adhesion to endothelial cells compared with WT mice (Fig 8B).

## Discussion

Our studies reveal that LPS disrupts circadian rhythms in marophages and blunts the expression of the core clock gene, *Bmal1* via a mechanism initiated by TLR4-dependent signaling. The ability of LPS to alter circadian rhythms is prevented by scavengers of superoxide and elevated superoxide levels are sufficient to impair transactivation of a circadian gene promoter by the core clock transcription factors, BMAL1 and CLOCK and promote a phase shift in circadian timing in U2OS cells. Macrophages from *Bmal1*-KO mice, which are unique from other circadian clock mutant mice in that they lack a functionally redundant isoform [10], have increased production of pro-inflammatory ROS, less NO, enhanced ability to take up cholesterol and increased migration and adhesion. Collectively these results support the concept that pro-inflammatory stimuli such as LPS can disrupt circadian rhythms in macrophages and that the loss of circadian regulation in macrophages can exaggerate a proinflammatory phenotype that could contribute to the eventual development of cardiovascular disease.

In isolated macrophages we found that LPS, via TLR4, induced a phase shift in circadian rhythms and suppressed the peak expression of a core clock gene, *Bmal1*. This data is consistent with that of others showing that LPS alters circadian timing and the expression of clock genes in intact animals [34, 56], isolated lungs [57], rhythmic cytokine release in macrophages [24] and more recently that LPS promotes a reduction in the expression of the master regulator of circadian transcription, *Bmal*1 and also *Per2* in macrophages from both animal models and humans [33]. Our assessment of Bmal1 mRNA revealed a 16h cycle time instead of the expected 24h oscillation which may be a result of the low resolution of sampling (3 time points per 24h), but this precludes any conclusions about the ability of LPS to alter circadian rhythmicity of *Bmal1* mRNA.”. The effects of LPS on circadian function were suppressed by LPS-RS a form of LPS from *Rhodobacter sphaeroides* that is hypoacylated and antagonizes the actions of LPS at TLR4 receptors. In addition to its actions on cell surface receptors, LPS has been recently shown to have intracellular actions by direct binding to caspase-4, a mechanism that is also inhibited by LPS-RS [58]. Whether caspase-4-dependent intracellular signaling contributes to the ability of LPS to disrupt circadian rhythms and alter inflammatory signaling is not yet known. LPS is also unlikely to be the only proinflammatory stimuli that can disrupt circadian rhythms. Other stimuli such as TNFα have been shown to alter circadian rhythms [59] and chronic inflammation, such as observed in cardiovascular disease and diabetes may also play a significant role [60, 61]. We also observed an ability of low dose LPS to modestly decrease both circadian amplitude and acrophase compared to the control and higher doses of LPS. The mechanisms underlying these differences are not known and it is possible they are a continuum of the same mechanisms seen in higher doses of LPS. While rhythm ablation and its impact have been well described, the impact of amplitude changes with regard to circadian biology are much less well-understood.

In macrophages, LPS is a well-established stimulus for superoxide [62] and NO [63] synthesis. In our study we found that the production of superoxide, but not NO, was necessary for disruption of circadian rhythms in macrophages and sufficient in U2OS cells. This observation is consistent with studies reporting that reactive oxygen species (ROS) can affect circadian timing in other organisms [64, 65] but is in contrast to data in mice with heterozygous deletion of SOD1, where elevated ROS did not affect circadian patterns of activity [66]. The reasons for this remain unclear but may relate to the level of ROS or the local environment or the cell type where the ROS is being produced. Other studies have shown similar inconsistencies with NO, with some reports indicating that the loss of endothelial NO does not influence blood pressure rhythms [67] which contrasts studies showing that NO can influence both blood pressure rhythms [68] and circadian timing in the SCN [69]. In our study, one explanation for the failure of NO to alter circadian rhythms in macrophages and U2OS cells may be the relative lack of expression of NO-dependent signaling molecules such as sGC/PKG which may be more abundantly expressed in the vasculature and the SCN [70]. The mechanism by which superoxide alters circadian rhythms is not yet known. It has been shown that LPS induces the expression of miR-155 in macrophages which silences *Bmal1* expression and alters circadian rhythm [33]. Whether superoxide contributes to the increased expression of miR-155 is not yet known. Increased superoxide could also alter cell signaling, phosphatases and redox balance which may affect clock timing. With regard to the latter, a close relationship exists between cellular metabolism, redox state and the circadian clock. The synthesis of NADPH is rhythmic and the binding of BMAL1 and its partners to DNA is strongly enhanced in the presence of NADPH or NADH and inhibited by NADP+ and NAD+ [71]. It is possible that in the presence of high levels of ROS, changes in redox balance, lower levels of NADPH and NADH and higher NADP+ and NAD+, contribute to the circadian disruption observed in macrophages.

It has been shown that disruption of circadian rhythms enhances the response to LPS in macrophages [23, 33]. Several components of the circadian clock including BMAL1-CLOCK and REV-ERBα have been linked to the NF-kB signaling cascade and inflammation [72, 73] and it is therefore not a completely surprising observation that disruption of circadian rhythms leads to greater inflammation. This concept is further supported by studies showing that transplantation of aortic grafts from *Bmal*1 knockout (KO) into littermate control WT mice is accompanied by robust arteriosclerosis and the up-regulation of macrophages in transplanted vessels [12]. *Clock*/ApoE double knockout mice also exhibit increased atherosclerosis and lipid laden macrophages compared to atherosclerosis prone mice with a functional clock [13]. Moreover, innate immune responses of macrophages can be controlled by micro-RNAs targeting *Bmal1* [33]. BMAL1 is a negative regulator of NF-kB signaling that binds to and restrains the acetylase activity of CLOCK. In the current study, we show that the loss of circadian rhythms in macrophages promotes an enhanced inflammatory phenotype with increased cytokine expression, increased ROS, reduced NO and greater cell adhesion and migration. These effects were observed in 2 distinct mouse models of circadian disruption, the *Bmal1* and *Per-*TKO indicating that deletion of both the positive and negative limbs of the clock yields a consistent phenotype. We have previously reported that clock-deficient mice have increased vascular production of ROS and reduced NO signaling [74]. Elevated ROS and reduced NO is a common observation in cardiovascular disease [42, 43] and is thought to contribute to a greater inflammatory response in macrophages through potentiation of NF-kB signaling and cytokine production [75, 76]. The primary source of superoxide in macrophages is gp91phox/NOX2 and we found that macrophages from circadian knockout mice have increased basal and LPS-stimulated expression of NOX2 which is consistent with the increased ability to produce ROS. We also observed increased expression of *Tnfα* and *Il6* in macrophages from *Bmal1* and *Per-TKO* mice. This is in agreement with other studies showing that CRY- and BMAL1- deficient macrophages exhibit a marked increase in TNFα and IL-6 compared with wild-type macrophages [33, 77] and suggests that clock function in macrophages is necessary to maintain an anti-inflammatory phenotype.

The circadian clock plays an important role in the development of cardiovascular disease and global disruption of clock function in genetically modified mice results in endothelial dysfunction [74], diabetes [78] and obesity [79], atherosclerosis [13, 80] and transplant arteriosclerosis [12, 13]. All of these cardiovascular diseases and associated disorders have important inflammatory components and while high levels of cholesterol and glucose are associated with the development of cardiovascular disease, it is the level of systemic inflammation that best predicts the risk of cardiovascular disease [81]. The intravascular infiltration of macrophages is well established to play a critical role in plaque formation in atherosclerosis and other cardiovascular disease [82, 83]. It is well established that LPS stimulates inflammation in macrophages and alters macrophage behavior resulting in greater ROS, lipid uptake, migration and adhesion [84–86] and that all of these attributes are thought to underlie the ability of LPS to enhance atherosclerosis [87]. These pro-atherogenic effects are mediated by the cellular receptor for LPS, TLR4 and loss of this receptor in TLR4, ApoE double knockout mice results in reduced atherosclerosis [88]. Despite substantial evidence for a prominent role of the circadian clock in regulating numerous aspects of the immune system, the importance of the macrophage intrinsic circadian clock and changes in clock function in response inflammatory signals such as LPS to the development of cardiovascular disease remains incompletely understood. Our data shows that in addition to regulating inflammation in macrophages, the circadian clock can influence the ability to take up LDL. We found that the loss of circadian rhythms results in increased cholesterol uptake and this data is consistent with other observations demonstrating clock-dependent regulation of LDL uptake. In *Rev-Erbα* KO mice or REV-ERBα knockdown in hypercholerolemic mice, (models where REV-ERBα repression would be expected to increase BMAL1, given their role as transcriptional repressors of *Bmal*1) LDL levels are increased in response to LPS [85]. While in *Clock* mutant mice crossed to hypercholesterolemic mice, LDL uptake is increased in macrophages [13]. However, these studies did not assess the effect on ROS or inflammation.

In addition to the roles of circadian clock genes in regulating the timing of gene expression, they have other functions that have been referred to as “off-clock” actions that are not directly related to transactivation of genes that undergo rhythmic cycling. For example, *Clock* can function as an acetyltransferase that acetylates histones and other proteins including p65 [11, 12]. The binding of BMAL1 with CLOCK promotes the transactivation of genes subject to circadian regulation but in turn limits the acetylation of p65 which is required for NFκB signaling. The loss of BMAL1 is therefore thought to promote increased inflammation via enabling CLOCK to acetylate p65 and facilitate NFκB signaling [13]. However, as the expression level of BMAL1 is also subject to oscillation, the separation of “off clock” effects of circadian clock genes from their roles in regulating circadian timing is challenging. A role for BMAL1 in the development of atherosclerosis was recently shown where the constitutive loss of BMAL1 expression increases lesion size in genetically susceptible mice [14]. Surprisingly, however, the inducible loss of BMAL1 expression, post-development, did not increase atherosclerosis. These findings suggest that BMAL1 may have unique and perhaps “off clock” roles during development. However, given the robustness of the circadian clock, residual BMAL1 expression that escapes the inducible disruption, may be sufficient to maintain clock function in select cells during light:dark conditions. Indeed robustness of the clock has been shown in other studies where circadian clock timing is maintained under a variety of genetic (clock gene depletion) and environmental stressors and multiple compensatory mechanisms underlie the robustness of the clock [89]. That ROS are able to impact clock function in macrophages may reflect an ability to disrupt multiple components of the clock.

Herein, our data serves to demonstrate a novel role for superoxide in regulating the timing of the circadian clock which contributes to macrophage activation and the inflammatory response. Loss of circadian rhythms further biases the macrophage phenotype towards pro-inflammatory behaviors that include increased superoxide, cytokine expression, cell adhesion and migration and cholesterol uptake. Together these results suggest a feedforward circuit by which inflammatory stimuli alter circadian timing to elicit a more pronounced pro-inflammatory phenotype. These findings further suggest that repetitive stressors to circadian timing, particularly for those in occupations such as working night shifts or with long distance travel, may surreptitiously increase inflammation. It will be important in future studies to determine if mice with the selective loss of circadian rhythms in macrophages impacts the development of cardiovascular disease.
